# Immuntherapien zur Behandlung der chronischen Hepatitis-B-Virusinfektion – eine Übersicht unter besonderer Berücksichtigung von CAR-T-Zellen

**DOI:** 10.1007/s00103-020-03223-7

**Published:** 2020-09-29

**Authors:** Zoltan Ivics, Maximilian Amberger, Tobias Zahn, Eberhard Hildt

**Affiliations:** 1 Abteilung Biotechnologie, Paul-Ehrlich-Institut, Langen, Deutschland; 2 Abteilung Virologie, Paul-Ehrlich-Institut, Paul-Ehrlich-Str. 51–59, 63225 Langen, Deutschland

**Keywords:** Hepatitis-B-Virus, Immuntherapie, CAR-T-Zellen, Checkpointinhibitoren, Impfstoffe, Hepatitis B virus, Immune therapy, CAR T cells, Checkpoint inhibitors, Vaccines

## Abstract

Derzeit leiden weltweit mehr als 250 Mio. Menschen an einer chronischen Infektion mit Hepatitis-B-Virus (CHB). Eine chronische Infektion geht mit einem erhöhten Risiko der Entwicklung einer Leberfibrose/-zirrhose und der Entwicklung eines hepatozellulären Karzinoms einher. Derzeit versterben jährlich ca. 0,8–1 Mio. Menschen an den Folgen einer chronischen Infektion. Eine Schwierigkeit bei der Therapie der CHB besteht darin, dass das virale Genom in Form eines Minichroms sehr lange Zeit persistieren kann bzw. dass virale Sequenzen in das Wirtsgenom inserieren können. Chronische Infektionen sind häufig durch funktionale Defekte der zellulären Immunantwort, insbesondere der T‑Zell-Antwort charakterisiert, was einer Eliminierung HBV-infizierter Zellen entgegensteht.

Immuntherapien zur Heilung der CHB zielen daher darauf ab, die antivirale Funktion der zellulären Immunantwort wiederherzustellen. Im Rahmen dieser Übersicht sollen verschiedene aktuelle Ansätze zur Immuntherapie der CHB beschrieben werden, insbesondere gentechnisch veränderte autologe T‑Zellen als mögliches Werkzeug zur Therapie der CHB. Weiterhin werden die Modulation von Checkpointinhibitoren der Immunantwort, metabolische T‑Zelltherapien und die therapeutische Impfung zur Stimulation der T‑Zellantwort als immuntherapeutische Strategien zur Therapie der chronischen HBV-Infektion zusammenfassend dargestellt.

## Abstract (en)

More than 250 million people worldwide suffer from chronic infection with hepatitis B virus (CHB). Chronic infection is associated with an increased risk of developing liver fibrosis/cirrhosis and hepatocellular carcinoma. Approximately 0.8–1 million people die annually as a result of CHB. One difficulty in the treatment of CHB is that the viral genome can persist for a very long time in the form of a minichromosome, and viral sequences can insert themselves into the host genome. Chronic infections are often characterized by functional defects of the cellular immune response, especially the T‑cell response, which prevents the elimination of HBV-infected cells.

Immunotherapies aiming to cure CHB therefore aim to restore the antiviral function of the cellular immune response. In this review, various current approaches to immunotherapy of CHB are described, in particular the use of genetically modified autologous T cells as a possible tool for therapy. Furthermore, the modulation of checkpoint inhibitors of the immune response, metabolic T cell therapies, and therapeutic vaccination to stimulate the T‑cell response are summarized as immunotherapeutic strategies for treating CHB.

## Humanes Hepatitis-B-Virus und Infektionsprozess

Das humane Hepatitis-B-Virus (HBV) gehört zur Familie der Hepadnaviren. Die Hepadnaviren umfassen einerseits die Orthohepadnaviren: WHV („woodchuck hepatitis virus“), GSHV („ground squirrel hepatitis virus“) und WMHBV („woolley monkey hepatitis B virus“) und andererseits die avianen Hepadnaviren: DHBV („duck hepatitis B virus“), HHBV („heron hepatitis B virus“) und STHBV („stork hepatitis B virus“). Charakteristisch für die Hepadnaviren sind eine hohe Gewebsspezifität, es werden nahezu ausschließlich Hepatozyten infiziert, und eine ausgeprägte Speziesspezifität. So infiziert HBV ausschließlich Menschen und Schimpansen. Menschen sind nicht permissiv für andere Hepadnaviren [1].

HBV ist ein umhülltes Virus. Die Hülle wird aus den 3 Hüllproteinen LHBs („large hepatitis B virus surface antigen“), MHBs („middle hepatitis B virus surface antigen“) und SHBs („small hepatitis B virus surface antigen“) aufgebaut und umgibt das Nucleocapsid, das aus dem Core-Protein aufgebaut ist. Das Nucleocapsid beherbergt das partiell doppelsträngige zirkuläre ca. 3,2 kB große DNA-Genom. Das HBV-Genom umfasst 4 verschiedene offene Leserahmen. Diese codieren für die HBV-Polymerase (P), die viralen Hüllproteine (LHBs, MHBs und SHBs – in ihrer Gesamtheit als HBsAg bezeichnet), das regulatorische X Protein (HBx) sowie für das Capsid (HBcAg) bzw. dessen sekretorische Variante (HBeAg).

Nach der Bindung von HBV an einen Rezeptor(-komplex), hier konnte der natrium-(Na^+^-)abhängige Gallensalztransporter NTCP als Rezeptormolekül identifiziert werden, erfolgt die Internalisierung über rezeptorvermittelte Endozytose. Im weiteren Verlauf des Infektionsprozesses erfolgt ein gerichteter Transport des Nucleocapsids zum Kernporenkomplex, wo dieses zerfällt und das partiell doppelsträngige zirkuläre DNA-Genom, welches kovalent mit der viralen Polymerase verknüpft ist, in den Zellkern transportiert wird [2–4]. Ein bipartites Kernlokalisationssignal (NLS) in P vermittelt den Import des mit der Polymerase verknüpften Genoms in den Zellkern. Dort erfolgt die Umwandlung des partiell doppelsträngigen zirkulären Genoms (RC Relaxed Circular) in ein komplett doppelsträngiges, kovalent geschlossenes, zirkuläres Genom (cccDNA, „covalently closed circular DNA“). Die cccDNA kann mit Histonen der Wirtszelle komplexiert werden und kann solcherart stabilisiert über lange Zeit als Minichromosom persistieren [5, 6]. Weiterhin können HBV-Genomfragmente in das Genom des Wirts integrieren. Nahezu alle HBV-assoziierten HCCs (hepatozelluläre Karzinome) weisen integrierte DNA auf, der eine wesentliche Rolle bei der virusassoziierten Pathogenese zugeschrieben wird.

Ausgehend von der cccDNA können nun verschiedene Transkripte gebildet werden [7]. Initial im Zuge der HBV-Replikation entsteht eine ca. 3,5 kB große übergenomische RNA, die für die virale Polymerase und HBcAg codiert. Die von diesem Transkript ausgehend translatierte Polymerase erkennt im Zytoplasma ein Verpackungssignal auf der prägenomischen RNA (Epsilonsignal) und rekrutiert dann Core-Proteine. Nachfolgend kommt es zum Zusammenbau eines Nucleocapsids, das die virale RNA beinhaltet. Durch die Reverse-Transcriptase-(RT-)Aktivität der viralen Polymerase wird das 3,5 kB große RNA-Prägenom wieder in das ca. 3,2 kB große DNA-Genom in seiner partiell doppelsträngigen zirkulären Form (RC-DNA) umgewandelt. Dieser Schritt stellt einen wesentlichen Angriffspunkt für antivirale Therapien dar, indem mittels Nucleosid/Nucleotid-Analoga die RT-Funktion von P gehemmt wird. Im weiteren Verlauf der Infektion wandert das neu gebildete DNA-haltige Nucleocapsid wieder an den Kernporenkomplex und der oben beschriebene Zyklus beginnt von Neuem. Dieser auf den ersten Blick widersinnige Zyklus dient dazu, den Spiegel an cccDNA im Zellkern zu erhöhen. Im weiteren Verlauf der Infektion entstehen dann neben den prägenomischen Transkripten übergenomische Transkripte (2,4 kB RNA, 2,1 kB RNA und 0,7 kB RNA), die für die Oberflächenproteine und für HBx codieren [8].

HBV-replizierende Zellen setzen neben den infektiösen viralen Partikeln noch große Mengen an subviralen Partikeln frei, die ausschließlich aus HBsAg aufgebaut sind, kein Genom enthalten und somit nicht infektiös sind. Hierbei werden 2 Formen unterschieden, zum einen die ca. 22 nm großen Sphären, die ganz überwiegend aus SHBs aufgebaut sind und geringe Mengen an LHBs und MHBs aufweisen, und die Filamente, die im Vergleich zu den Sphären einen deutlich größeren Anteil an LHBs aufweisen. Weiterhin setzen HBV-replizierende Zellen mit Ausnahme von HBV-Genotyp‑G eine sekretorische Variante des Core-Proteins frei, das HBeAg, das einen wesentlichen diagnostischen Marker darstellt.

## Chronische HBV-Infektion

Weltweit haben aktuell ca. 2.000.000.000 Menschen eine HBV-Infektion durchgemacht, trotz des Vorhandenseins wirksamer Impfstoffe. HBV ist eine sexuell übertragbare Krankheit, die auch durch kontaminierte Blutprodukte übertragen werden kann. Eine hohe Verbreitung von HBV ist insbesondere in zahlreichen Ländern der Dritten und Vierten Welt zu beobachten, was auch dadurch bedingt ist, dass die sozioökonomischen Verhältnisse bisher eine Impfung breiter Bevölkerungsschichten nicht ermöglicht haben. Die Häufigkeit der Chronifizierung der Infektion hängt vom Lebensalter der Infizierten ab. Eine Infektion unter der Geburt oder im frühen Kindesalter führt häufiger zu einer chronischen Infektion als im Erwachsenenalter. Derzeit sind weltweit ca. 250 Mio. Menschen chronisch mit HBV infiziert [9, 10]. Die chronische Infektion kann verschiedene Verlaufsformen nehmen. So kann bspw. zwischen dem gesunden Trägerstatus häufig mit großen Mengen an HBsAg und viralen Partikeln im Blut bei gleichzeitiger Abwesenheit klinischer Symptome oder der chronisch entzündlichen Hepatitis mit permanenter Schädigung des Lebergewebes unterschieden werden. Daneben sind auch sog. okkulte Formen beschrieben mit minimalen Mengen an HBV-DNA und HBsAg-Mengen, die unter dem Detektionslimit liegen. Auf der Basis des serologischen Profils und der Entzündungsmarker der Leber können 5 Phasen unterschieden werden, (i) HBeAg-positive chronische HBV-Infektion, (ii) HBeAg-positive chronische Hepatitis, (iii) HBeAg-negative chronische Hepatitis, (iv) HBeAg-negative chronische Infektion und (v) HBsAg-negative okkulte HBV-Infektion charakterisiert durch Anti-HBc [11].

Eine chronische HBV kann abhängig von der Verlaufsform zu einer Leberfibrose, -zirrhose oder einem hepatozellulären Karzinom (HCC) führen. So versterben derzeit weltweit ca. 0,8 bis 1 Mio. Menschen pro Jahr an den Folgen einer chronischen HBV-Infektion und HBV ist eine der Hauptursachen für die Entstehung eines HCCs [12].

Die Therapie der chronischen HBV-Infektion basiert derzeit in erster Linie auf der Gabe von Nucleos(t)idanaloga der dritten Generation, welche die RT-Aktivität der HBV-Polymerase hemmen. Dabei kommt es jedoch nur in weniger als 10 % der Fälle zum dauerhaften Verlust der HBsAg-Bildung, was eine langfristige Therapie erforderlich macht und das Risiko der Reaktivierung birgt. Ursächlich hierfür sind die hohe Stabilität der als Minichromosom vorliegenden cccDNA und die Expression HBV-spezifischer Sequenzen ausgehend von integrierter HBV-DNA. Alternativ dazu kann eine Therapie basierend auf Interferon-Alpha-Gaben erfolgen, die jedoch mit Nebenwirkungen und somit einer geringen Compliance einhergehen kann [13].

Die Ursachen für die Persistenz einer HBV-Infektion und die damit verbundene Entwicklung einer chronischen HBV-Infektion (CHB) sind nicht vollständig verstanden. Die Immunantworten bei einer CHB sind häufig charakterisiert durch Dysfunktion und Erschöpfung HBV-spezifischer CD4+- und CD8+-T-Zellen und/oder verminderte Anzahl und Dysfunktion von dendritischen Zellen (DCs) sowie von NKs/NKTs („natural killer cells“/„natural killer T cells“). Weiterhin wird häufig die verstärkte Expression von Checkpointproteinen wie PD‑1, CTLA‑4 und Tim‑3 beobachtet sowie eine gehemmte innate Immunantwort, häufig charakterisiert durch Dysfunktion und verminderte Expression von Toll-like-Rezeptoren (TLR; [1, 9, 14, 15]).

In einem Circulus vitiosus bedingen inflammatorische Prozesse der Leber, dass es zu einer eingeschränkten Funktion der T‑Zellen kommt, um so die Integrität des Lebergewebes zu erhalten. Wesentliche Faktoren dabei sind die Freisetzung von Arginase und IDO aus den zerstörten Hepatozyten, die eine Depletion einzelner Aminosäuren bedingen, die essenziell für den Erhalt der T‑Zellfunktionalität sind. So bedingt eine Verminderung des Argininspiegels eine geringere CD3zeta-Menge in T‑Zellen, was letztlich zu einer gestörten Funktion TCR-abhängiger Signalwege führt [16].

Weiterhin kommt es durch die intrahepatische Inflammation zu einer verstärkten Rekrutierung regulatorischer T‑Zellen (Treg) und Aktivierung von Stellate-Zellen, was eine verstärkte Bildung von TGF-beta und IL10 bedingt.

**Figure 1:**
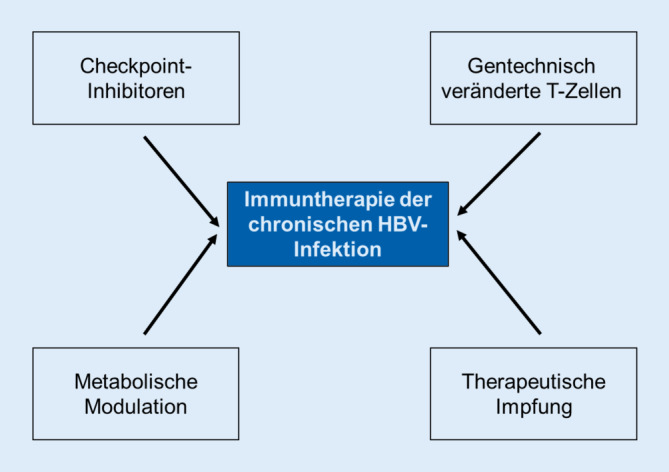

Auf der Basis dieser Erkenntnisse basieren viele derzeitige Therapieansätze der CHB auf der Wiederherstellung und Stimulation der antiviralen T‑Zellantwort. In dieser Übersichtsarbeit wird hierbei insbesondere auf gentechnisch veränderte autologe T‑Zellen, insbesondere Chimeric-Antigen-Receptor-(CAR-)T-Zellen als mögliches Werkzeug zur Therapie der CHB eingegangen, die daher zunächst ausführlicher eingeführt werden. Ein besonderer Fokus wird hierbei auf CAR-T-Zellen gelegt, da dies ein derzeit besonders intensiv bearbeitetes Forschungsfeld darstellt. Weiterhin werden die Modulation von Checkpointinhibitoren der Immunantwort auf metabolische T‑Zelltherapien und die therapeutische Impfung zur Stimulation der T‑Zellantwort beschrieben (Abb. 1; [17–19]). Eine Übersicht über derzeit laufende klinische Studien auf dem Gebiet der Immuntherapie von chronischen HBV-Infektionen kann unter https://clinicaltrials.gov gefunden werden.

## CAR-T-Zellen

**Figure 2:**
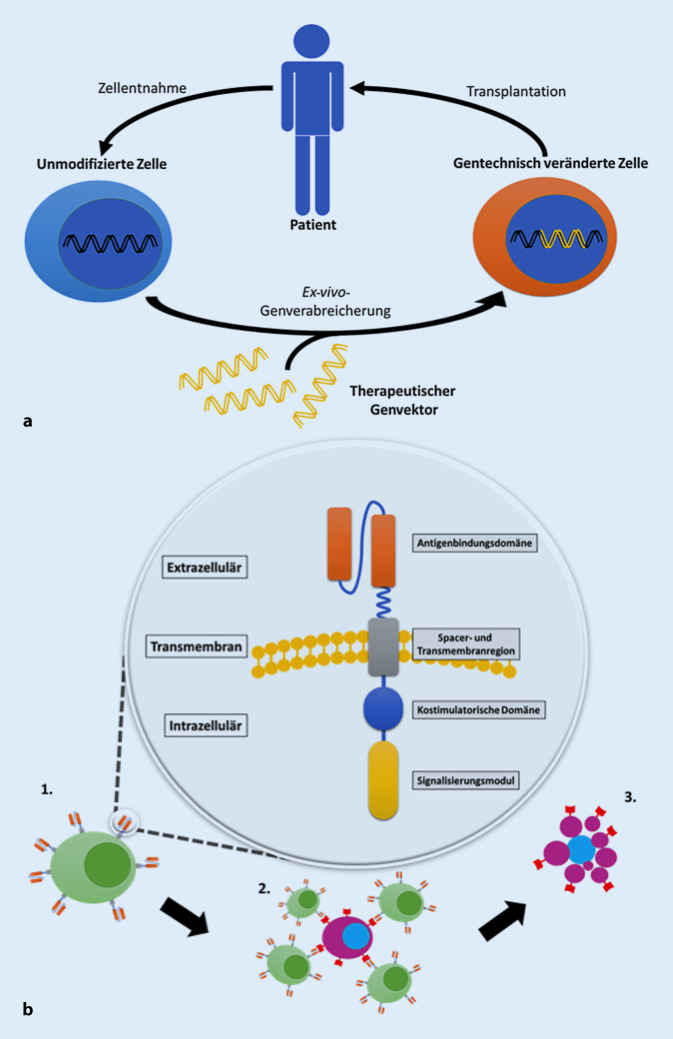

Wegen der derzeitigen Entwicklungen auf dem Gebiet der CAR-T-Zellen wird hier an dieser Stelle etwas ausführlicher darauf eingegangen. Bei der Ex-vivo-Genverabreichung wird der therapeutische Genvektor in eine ausgewählte Zellpopulation, die von einem Spender isoliert wurde, eingebracht und anschließend die gentechnisch veränderten Zellen in einen Patienten transplantiert (Abb. 2a). Die adoptive Immuntherapie mit T‑Zellen, die durch Gentransfer zur Expression eines synthetischen chimären Antigenrezeptors (CAR) manipuliert werden, entwickelt sich zu einer wirksamen und potenziell kurativen Behandlung von fortgeschrittenen Malignomen. CARs sind Designermoleküle, die sich aus verschiedenen Komponenten zusammensetzen: einer extrazellulären Antigenbindungsdomäne, meist den variablen leichten und schweren Ketten eines monoklonalen Antikörpers (scFv), einer Spacer- und Transmembranregion, die den Rezeptor auf der T‑Zelloberfläche verankert und Reichweite und Flexibilität für die Bindung des Zielepitops bietet, und einem intrazellulären Signalisierungsmodul, meist CD3zeta und einer oder mehreren kostimulatorischen Domänen, die die T‑Zellaktivierung nach der Antigenbindung [20, 21] vermitteln (Abb. 2b), was zur selektiven Abtötung der Zielzelle führt.

Mehrere klinische Studien haben gezeigt, dass humane CAR-T-Zellen gegen den B‑Zelllinienmarker CD19 (CD19+-CAR-T-Zellen) in der Lage sind, dauerhafte vollständige Remissionen bei Patienten mit chemotherapie- und strahlentherapierefraktärer B‑zellakuter lymphatischer Leukämie (B-ALL), Non-Hodgkin-Lymphom (NHL) und chronischer lymphatischer Leukämie (CLL; [22–28]) zu induzieren. Angesichts des fortgeschrittenen Krankheitsstadiums und des Versagens konventioneller Behandlungen bei vielen Patienten, die in diese klinischen Studien eingeschlossen wurden, werden diese Ergebnisse von vielen als ein medizinischer Durchbruch in der Tumortherapie angesehen. Die Nebenwirkungen der CD19+-CAR-T-Zelltherapie sind eine Folge der starken Antitumorimmunreaktion und umfassen das Tumorlysesyndrom aufgrund der raschen Zerstörung einer großen Anzahl von Tumorzellen, das Zytokinfreisetzungssyndrom aufgrund der raschen Freisetzung von Zytokinen durch CAR-T-Zellen und andere Immunzellen, die Neurotoxizität, wahrscheinlich als Folge einer entzündungsbedingten endothelialen Dysfunktion und einer erhöhten Blut-Hirn-Schranken-Permeabilität, sowie die Depletion normaler B‑Zellen aufgrund ihrer physiologischen Expression von CD19 [29, 30]. Zwei CAR-T-Zellprodukte haben in den USA und in der Europäischen Union die Marktzulassung für die Behandlung von CD19+ ALL und NHL erhalten und sorgten beide aufgrund ihres beträchtlichen Marktpreises und der komplexen Logistik hinter dieser Behandlung für Schlagzeilen.

**Figure 3:**
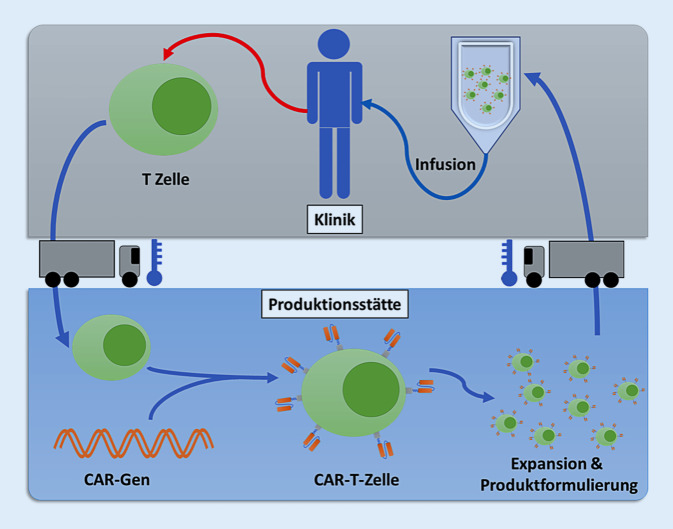

Die Herstellung von CAR-T-Zellen umfasst die Entnahme der T‑Zellen des Patienten, den Transport zu einer zentralen Produktionsstätte, um den CAR-Gentransfer und die T‑Zellexpansion durchzuführen, sowie den Rücktransport des kryokonservierten Zellprodukts in das Krankenhaus, wo dann die Therapie durchgeführt wird (Abb. 3). Dieses Verfahren stellt zweifellos eine komplexe Logistik dar, die derzeit mit erheblichen Kosten und Wartezeiten verbunden ist. Darüber hinaus sind die Herstellungskapazitäten spezialisierter Good-Manufacturing-Practice-(GMP‑)Produktionsanlagen derzeit limitiert.

Die transiente CAR-Expression in T‑Zellen durch Transfektion von mRNA führt zu einer bis zu mehrtägigen CAR-Oberflächenexpression, was ausreichen kann, um einen Antitumoreffekt zu induzieren, insbesondere wenn hohe Dosen von CAR-T-Zellen verabreicht werden [31, 32]. Wegen des raschen Rückgangs der CAR-Expression sind jedoch mehrere aufeinanderfolgende Verabreichungen erforderlich, um den therapeutischen Effekt aufrechtzuerhalten.

Bei der überwältigenden Mehrheit der präklinischen Arbeiten und klinischen Studien, die über die Wirksamkeit der CD19-CAR-T-Zelltherapie berichteten, wurden gammaretrovirale und lentivirale Vektoren eingesetzt, um die genetische Information des CD19-CAR stabil in die T‑Zellen der Patienten zu integrieren. Infolgedessen wird der CAR nicht nur in T‑Zellen exprimiert, die modifiziert und dem Patienten infundiert werden, sondern auch in nachfolgenden Generationen von Tochter-T-Zellen nach der Zellteilung und -vermehrung. Die Verwendung stabiler Gentransfersysteme steht im Einklang mit dem Konzept und fördert das Bestreben der CAR-T-Zellimmuntherapie, bereits mit der Verabreichung einer kleinen Anzahl von patienteneigenen CAR-T-Zellen ein Anwachsen und Proliferation in vivo zu erreichen, bis alle Tumorzellen entfernt sind, sowie die Persistenz der Gedächtnis-CAR-T-Zellen, die den Patienten langfristig vor einem Tumorrezidiv schützen.

Letztlich sind transposonbasierte, nichtvirale Gentransfertechnologien auch für die Herstellung von CAR T‑Zellen anwendbar [33–35]. Ähnlich wie virale Technologien ermöglichen Transposons die genomische Integration von CAR-Transgenen, aber im Gegensatz zu viralen Vektoren werden die Komponenten von Transposonvektoren als nackte Nukleinsäuren hergestellt und als solche in die Zielzellen eingebracht. Das Potenzial der Sleeping-Beauty-(SB‑)Transposition [36] zur Integration der genetischen Information des CAR in T‑Zellen wurde zuerst von Cooper et al. [37] erforscht. Es wurde gezeigt, dass die SB-Transposase entweder als Plasmid-DNA oder mRNA in Kombination mit einem plasmidcodierten CAR-Transposon bereitgestellt und durch Elektroporation in T‑Zellen eingeführt werden kann, um funktionelle CD19+-CAR-T-Zellen zu erhalten.

## Gentechnisch veränderte T-Zellen zur Therapie der CHB

Derzeitige T‑zellbasierte Therapien zielen darauf ab, in vitro funktionelle HBV-spezifische T‑Zellen zu generieren, die in den chronisch HBV-positiven Patienten reinfundiert werden. Dabei stehen CAR-T-Zellen- und T‑zellrezeptor-(TCR-)basierte T‑Zelltherapien im Vordergrund. Die Rationale für diese Ansätze basiert auf der Beobachtung, dass Knochenmarkstransplantationen von Patienten, die eine HBV-Infektion überwunden hatten, in Patienten mit einer CHB zu einem Verschwinden des HBsAg geführt haben. Im Falle der TCR-basierten Therapien werden aus dem jeweiligen CHB-Patienten zirkulierende T‑Lymphozyten isoliert und in vitro aktiviert und expandiert. Mittels viraler Vektoren oder Elektroporation erfolgt der Transfer HBV-spezifischer TCR-Gene. Die so modifizierten Zellen werden in den CHB-Patienten reinfundiert und sollen dort die HBV-positiven Zielzellen erkennen und lysieren. Das können einerseits HBV-positive Hepatozyten sein und andererseits auch aus einem HCC heraus metastasierende Zellen in extrahepatischen Geweben. Die HBV-spezifischen TCR-T-Zellen sind HLA-Klasse 1 restringiert, sodass HBV-spezifische Peptide nur im Kontext der passenden HLA-Moleküle erkannt werden können. Dies bietet den Vorteil, dass keine Hemmung durch das ggf. in großen Mengen zirkulierende HBsAg und HBeAg erfolgen kann [38, 39].

Eine Schwierigkeit hierbei besteht jedoch darin, dass es bei einer schlagartigen Eliminierung aller HBV-positiven Zielzellen zu einer fulminanten hepatitisanalogen Situation kommen kann. Dem kann einerseits durch eine sogfältig gestaffelte Dosierung Rechnung getragen werden oder durch eine Modifizierung der T‑Zellen, sodass geringere Mengen von Perforin und/oder Granzym B gebildet werden und somit das lytische Potenzial vermindert wird [40].

Eine Alternative dazu stellen CAR-T-Zellen dar. Die Funktion der CAR-T-Zellen hängt nicht vom HLA-Haplotypen des Patienten ab. HBV-spezifische CAR-T-Zellen tragen einen chimären Antigenrezeptor, der ein HBV-spezifisches Antikörperfragment mit einem kostimulatorischen CD28-Molekül und der intrazellulären Domäne von CD3zeta kombiniert. So konnten CAR-T-Zellen, die gegen die a‑Determinante von HBsAg oder aa 37–43 der PreS1-Domäne etabliert sind, die HBsAg-positiven Hepatozyten erkennen. Dabei wurde einerseits festgestellt, dass es zur spezifischen Lyse HBV-positiver Zellen kommen kann. Andererseits gibt es auch Berichte, dass nach adoptivem Transfer von CAR-T-Zellen der zweiten Generation in humanisierten Mäusen mit HBV-infizierten Hepatozyten zwar eine deutliche Verminderung der Viruslast und des HBsAg-Spiegels zu beobachten ist, jedoch die Marker für eine Leberzellschädigung und Leberfunktionalität währenddessen weitgehend unverändert blieben, was auf eine nonzytophatische Lyse hindeutet. Derzeit wird der HBV-spezifischen CAR-T-Zelltherapie bzw. TCR-T-Zelltherapie ein großes Potenzial in der Therapie der CHB zugeschrieben [17, 41].

Neben T‑Zellen stellen auch NK-Zellen („natural killer cells“) einen möglichen Ansatzpunkt für eine Immuntherapie dar. NK-Zellen kommt ebenfalls eine prominente Rolle bei der Kontrolle viraler Infektionen zu, da sie einerseits antivirale Zytokine produzieren und andererseits auf virusinfizierte Zellen zytotoxisch wirken können. In Patienten mit CHB scheint die zytolytische Aktivität zu überwiegen, während die Kapazität zur Produktion antiviraler Zytokine vermindert ist. Daneben üben in CHB-Patienten NK-Zellen häufig einen hemmenden Effekt auf virusspezifische T‑Zellen aus.

Im Bereich der Tumortherapie befinden sich bereits verschiedene Ansätze zur Modulation der NK-Aktivität in der präklinischen oder frühen klinischen Phase. Dazu gehören die Modulation des PD-1/PD-1L-Systems oder TIGIT, Tim-3- oder TGFbeta-Hemmung oder die Stimulation von NK-Zellen mit IL15, IL12 und IL18. Daneben gibt es auch erste Ansätze zur Herstellung von CAR-NK-Zellen. Die Übertragung dieser Ansätze auf die Therapie der CHB ist derzeit Gegenstand verschiedener experimenteller Forschungsprojekte.

## Checkpointinhibitoren

Die verstärkte Expression von Checkpointinhibitoren wurde erstmals im Zusammenhang mit LCMV-Infektionen (lymphozytäres Choriomeningitisvirus) beschrieben. Hier konnte auch beobachtet werden, dass die antivirale Funktion erschöpfter T‑Zellen durch die Hemmung inhibitorischer Pathways wieder rekonstituiert werden konnte. Für die CHB wurde eine deutlich erhöhte Expression von PD‑1 und 2B4 bei intrahepatischen Lymphozyten gefunden, wie auch eine verstärkte Expression von Checkpointliganden wie PD-L1 auf B‑Zellen und Monozyten oder von Galectin 9 auf Kupferzellen gefunden wurde. Obgleich bei In-vitro-Experimenten zu beobachten war, dass eine PD-1/PD-L1-Blockade zu einer partiellen Rekonstituierung der B‑ und T‑Zellfunktion führt und im WHV-Modell PD-L1-spezifische Antikörper in Kombination mit Entecavir- und DNA-Immunisierung einen deutlichen Einfluss auf die Infektion hatten, zeigte es sich, dass im Falle der CHB die alleinige Applikation von Checkpointinhibitoren nur eine sehr begrenzte Wirksamkeit hat, obgleich es Berichte über die Wiederherstellung der CD8-T-Zellantwort nach PD-1-Blockierung oder auch anderer Checkpointinhibitoren wie Tim‑3 und CTLA‑4 gibt. Allerdings zeigt sich, dass die Wirksamkeit dieses Ansatzes auch stark von einer Reihe von Wirtsfaktoren abhängen könnte. Ein besonderer Fokus liegt derzeit hierbei auf der Charakterisierung von DNA-Methylierungen, die auch nach PD-1-Blockade anhalten und so den Effekt der PD‑1 Inhibitoren konterkarieren könnten, sodass derzeitig die kombinierte Applikation von PD-1-Inhibitoren und DNA-demethylierenden Substanzen untersucht wird [42–44].

## Metabolische Modulation

Verschiedene Funktionen der T‑Zellen gehen mit unterschiedlichen metabolischen Erfordernissen einher und erfolgen an unterschiedlichen Orten aufgrund des Wanderns von T‑Zellen in verschiedene Gewebe. Somit hat der Metabolismus der Zellen einen wesentlichen Einfluss auf die Immunantwort. So kommt bspw. der Verminderung des Argininspiegels durch erhöhte Arginase-I-Aktivität, die aus zerstörten Hepatozyten oder von myeloiden Suppressorzellen (MDSC) freigesetzt wird, hierbei eine wesentliche Rolle bei der Modulation der T‑Zellaktivität zu. So kann mindestens teilweise die Aktivität CD8+-T-Zellen durch Erhöhung des Argininspiegels wieder erhöht werden. Weiterhin ist häufig auch eine Veränderung der Morphologie der Mitochondrien zu beobachten, die mit einer deutlich verminderten Expression einzelner Komponenten der Atmungskette und somit einer Depolarisierung der Mitochondrien und einer deutlich verstärkten Produktion von reaktiven Sauerstoffspezies (ROS) einhergeht. Spezifisch an den Mitochondrien wirkende Antioxidantien bedingen eine deutliche Verminderung des ROS-Spiegels, eine Wiederherstellung der mitochondrialen Depolarisierung und gehen mit einer nachfolgenden erhöhten Zytokinproduktion der HBV-spezifischen T‑Zellen einher. Somit kommt der Wiederherstellung der mitochondrialen Aktivität ebenfalls eine Rolle bei der Rekonstituierung der T‑Zellfunktion zu [45].

## Induktion HBV-spezifischer Immunantwort und therapeutische Impfstoffe

Während konventionelle HBV-Impfstoffe, die auf SHBs basieren, aufgrund der entwickelten Immuntoleranz keinen signifikanten therapeutischen Effekt bei einer CHB ausüben, konnte in HBV-exprimierenden Mäusen durch die Kombination von einer HBsAg-Vakzine mit IL12 als Adjuvans eine Überwindung der Toleranz beobachtet werden, die mit einer verstärkten CD8+/CD4+-T-Zellantwort einherging [46]. Ähnliche Ergebnisse konnten durch eine getrennte Gabe einer SHBs-basierten Vakzine und anschließender Immunisierung mit der PreS1-Domäne beobachtet werden [47].

Weitere experimentelle Ansätze in transgenen Mäusen basieren auf einer Kombination von HBsAg und HBcAg, adjuvantiert mit CpG-Oligonucleotiden, was neben der Induktion einer HBsAg/HBcAg-spezifischen B‑Zellantwort auch eine deutliche zelluläre Immunantwort auslöst, was zu einer Senkung des HBsAg-Spiegels führt ohne dass es zu einer Zerstörung der HBV-positiven Zellen kommt [48].

Weitere therapeutische Impfstoffe basieren aus Vektorplattformen. Hier wäre bspw. TG1050 zu nennen, die auf einer nichtreplikationskompetenten Adenovirus-5-Plattform basiert. Das adenovirale E1 ist durch ein Fusionsprotein aus einem modifizierten HBcAg, der viralen Polymerase und einzelnen HBsAg-Domänen aufgebaut. TG1050 wird derzeit im Rahmen einer Phase-1b-Studie untersucht. Ein weiterer Ansatz (TherVacB) basiert auf einer Prime-Boost-Strategie unter Verwendung von HBsAg- und HBcAg-Partikeln für das Priming, gefolgt von einer weiteren Immunisierung (Boost) durch einen MVA-basierten Vektor, der core-, preS-, S‑ und pol-spezifische Antigene codiert. TherVacB B ist derzeit noch in der präklinischen Phase [49].

Eine völlig neuartige Plattform basiert auf membranpermeablen HBV-Core-Partikeln, die als Antigenträger fungieren, welche über einen Adapter mit preS-spezifischen Antigenen beladen wurden. Aufgrund der hochgeordneten Struktur der Antigene auf der Capsidoberfläche kommt es zur robusten Induktion einer humoralen Immunantwort, während die Zellpermeabilität des Antigenträgers die effiziente Aufnahme in antigenpräsentierende Zellen (APCs) ermöglicht und so zur starken Induktion CD8-positiver T‑Zellen führt. Auch dieser Ansatz befindet sich derzeit noch in der präklinischen Phase [50].

Weitere Ansätze basieren auf einer Mischung HBV-spezifischer Peptide (HepTcellTM), die 9 verschiedene Peptide beinhaltet, die CD4- und CD8-T-Zellepitope umfassen. Dieser Ansatz wurde im Rahmen einer Phase-1-Studie untersucht in Verbindung mit Entecavir.

Ebenfalls in Verbindung mit Nucleosidanaloga wurden DNA-basierte Impfstoffe, wie INO-1800 (codiert auf pVax-Basis für S und Core) oder HB-110 (codiert für HBsAg, HBcAg und Pol), im Rahmen von Phase-1-Studien untersucht.

Grundsätzlich erscheint der Weg der therapeutischen Vakzinierung als Möglichkeit zur Heilung einer CHB, erfordert aber eine funktionelle Wiederherstellung der T‑Zellantwort (CD8 und CD4) sowie der B‑Zellantwort, um eine dauerhafte Kontrolle der HBV-Infektion ausüben zu können. Um das Problem der T‑Zellerschöpfung zu adressieren ist ggf. die Kombination mit Checkpointinhibitoren zu erwägen und eine Verminderung der Menge an zirkulierenden HBV-Antigenen vor der Immunisierung anzustreben. Eine wesentliche Bedeutung wird hierbei auch der Identifizierung von Wirtsfaktoren zukommen, welche für T‑Zellerschöpfung prädisponieren [44, 49, 51].
